# An ensemble approach for large-scale identification of protein-protein interactions using the alignments of multiple sequences

**DOI:** 10.18632/oncotarget.14103

**Published:** 2016-12-22

**Authors:** Lei Wang, Zhu-Hong You, Xing Chen, Jian-Qiang Li, Xin Yan, Wei Zhang, Yu-An Huang

**Affiliations:** ^1^ School of Computer Science and Technology, China University of Mining and Technology, Xuzhou 221116, China; ^2^ Xinjiang Technical Institute of Physics and Chemistry, Chinese Academy of Science, Urumqi 830011, China; ^3^ School of Information and Electrical Engineering, China University of Mining and Technology, Xuzhou 221116, China; ^4^ College of Computer Science and Software Engineering, Shenzhen University, Shenzhen, Guangdong 518060, China; ^5^ College of Information Science and Engineering, Zaozhuang University, Zaozhuang, Shandong 277100, China; ^6^ School of Foreign Languages, Zaozhuang University, Zaozhuang, Shandong 277100, China

**Keywords:** disease, position-specific scoring matrix, multiple sequences alignments, cancer

## Abstract

Protein–Protein Interactions (PPI) is not only the critical component of various biological processes in cells, but also the key to understand the mechanisms leading to healthy and diseased states in organisms. However, it is time-consuming and cost-intensive to identify the interactions among proteins using biological experiments. Hence, how to develop a more efficient computational method rapidly became an attractive topic in the post-genomic era. In this paper, we propose a novel method for inference of protein-protein interactions from protein amino acids sequences only. Specifically, protein amino acids sequence is firstly transformed into Position-Specific Scoring Matrix (PSSM) generated by multiple sequences alignments; then the Pseudo PSSM is used to extract feature descriptors. Finally, ensemble Rotation Forest (RF) learning system is trained to predict and recognize PPIs based solely on protein sequence feature. When performed the proposed method on the three benchmark data sets (*Yeast*, *H. pylori*, and independent dataset) for predicting PPIs, our method can achieve good average accuracies of 98.38%, 89.75%, and 96.25%, respectively. In order to further evaluate the prediction performance, we also compare the proposed method with other methods using same benchmark data sets. The experiment results demonstrate that the proposed method consistently outperforms other state-of-the-art method. Therefore, our method is effective and robust and can be taken as a useful tool in exploring and discovering new relationships between proteins. A web server is made publicly available at the URL http://202.119.201.126:8888/PsePSSM/ for academic use.

## INTRODUCTION

Protein–Protein Interactions (PPIs) play an important role in almost every cellular process [1, 2]. A variety of biochemical activities performed by PPIs are the foundation of life, such as immune response, regulation of transcription and translation, DNA replication, and endocrine function [3]. In recent decades, in order to understand the mechanisms of all kinds of biochemical activities, a variety of biological experimental methods have been designed to detect the interactions between proteins, for example, two-hybrid systems [4, 5], mass spectrometry [6, 7], immunoprecipitation [8], protein chip technology [9], etc. However, it is time-consuming, cost-intensive and small-scale to identify the interactions among proteins using biological experiments only. Therefore, there is an urgent need to use computational methods to predict protein-protein interactions efficiently and massively.

So far, a number of computational methods have been proposed to predict protein-protein interactions. These methods can be roughly divided into three types: structure-based methods [10–13], sequence-based methods [14–25] and function-annotation-based methods [26–29]. Among them, there is no need to know protein structure information and a pre-knowledge using the sequence-based approaches, which has aroused more and more interests in researchers. For example, Martin *et al.* developed a computational model to identify the interactions among proteins by using the signature descriptor [30]. This model achieved an accuracy of 70% and 80% when testing on the *H. pylori* and *Yeast* data sets by 10-fold cross-validation. Shen *et al.* proposed the conjoint triad approach to predict human PPIs considering the local environments of residues [16]. In the experiment, the accuracy of this model reached 83.9%. Ahmad *et al.* proposed an algorithm to predict the DNA-binding sites based on the neural network, which adopted amino acid sequences evolutionary information in terms of their position specific-scoring matrices [31].

In this paper, we propose a novel sequence-based computational method for predicting potential protein-protein interactions. Specifically, we first convert the protein amino acids sequence into the Position Specific Scoring Matrix (PSSM) [32] that contains the information of evolution; Then use the Pseudo Position-Specific Score Matrix (PsePSSM) [33–35] algorithm to extract features expecting more information. Finally, the Rotation Forest (RF) [36, 37] classifier is applied to determine whether the proteins are related or not. In the experiment, the proposed method is implemented on the *Yeast* data set, and the accuracy of five-fold cross-validation is 98%. At the same time, we also verified on the *Helicobacter. pylori, C.elegans*, *E.coli, H.sapiens* and *M.musculus* data sets, and yielded the accuracy of 89.75%, 98.50%, 91.00%, 97.45% and 98.08%, respectively. In order to further evaluate the prediction performance, we also compare the proposed method with other excellent methods. Comparison results show that the proposed method consistently outperforms other state-of-the-art methods.

## RESULTS AND DISCUSSIONS

### Evaluation measures

Four standard criteria are used to evaluate the performance of our approach, including accuracy (Accu.), sensitivity (Sen.), precision (Prec.) and Matthews correlation coefficient (MCC). MCC represents the correlation coefficient between the observed and the predicted class. It ranges from -1 (the best predictive model) to 1 (the worst predictive model). These measures are defined as follows:

$$Accu.=\frac{TP+TN}{TP+TN+FP+FN}$$ (1)

$$Sen.=\frac{TP}{TP+FN}$$ (2)

$$Prec.=\frac{TP}{TP+FP}$$ (3)

$$MCC=\frac{TP\times TN-FP\times FN}{\sqrt{\left(TP+FP\right)\left(TP+FN\right)\left(TN+FP\right)(TN+FN)}}$$ (4)

where TP denotes the number of positive samples to be correctly predicted; FP denotes the number of negative samples to be incorrectly predicted; TN denotes the number of negative samples to be correctly predicted; FN denotes the number of positive samples to be incorrectly predicted, respectively. In addition, the receiver operating characteristic (ROC) [38] curve is used to access the performance of classifier. In the ROC curve, the default threshold for the classifier is 0.5. The threshold will be changed with the true positive rate versus the false positive rate when a new set of prediction result is accepted; this change will be expressed through graphics.

### Assessment of prediction ability

**Figure 1:**
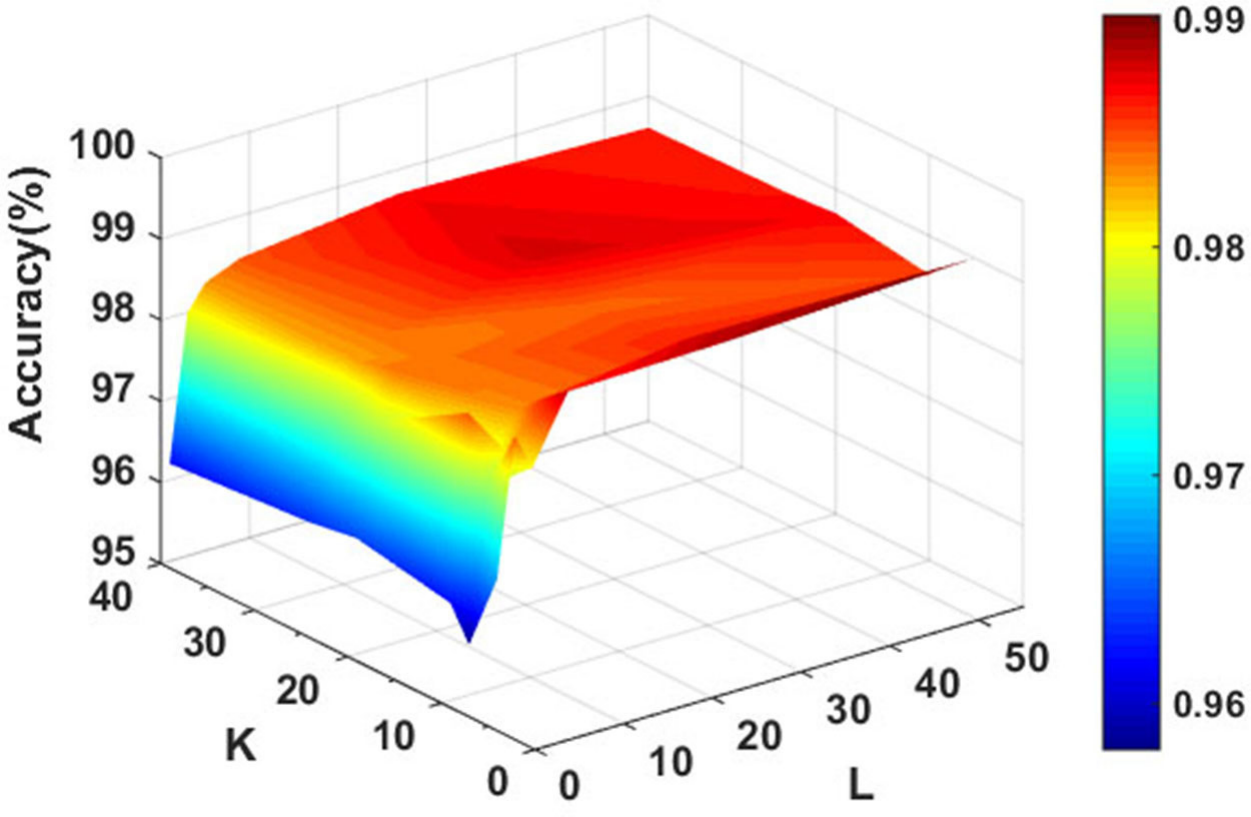
Accuracy surface obtained of rotation forest for optimizing regularization parameters *K* and *L*

In order to achieve the best performance of the rotation forest, we use the grid search method to adjust the corresponding parameters. In this study, PCA [36] was chosen as rotation forest transformation method and the J48 decision tree [39] derived from the WEKA machine learning workbench was selected as the base classifier. Figure 1 shows the accuracy of the classifier under different parameter values. From the Figure 1 we can see that our method performs well, the average prediction accuracy is rapidly increasing with the increase of the value of *L* at the beginning and increase rate becomes slow when the value of *L* is greater than 5. However, the accuracy always presents a fluctuation state with the increase of the value of the parameter *K*. After a comprehensive assessment, we choose the optimal parameters of *K=8* and *L=5* ultimately.

**Figure 2:**
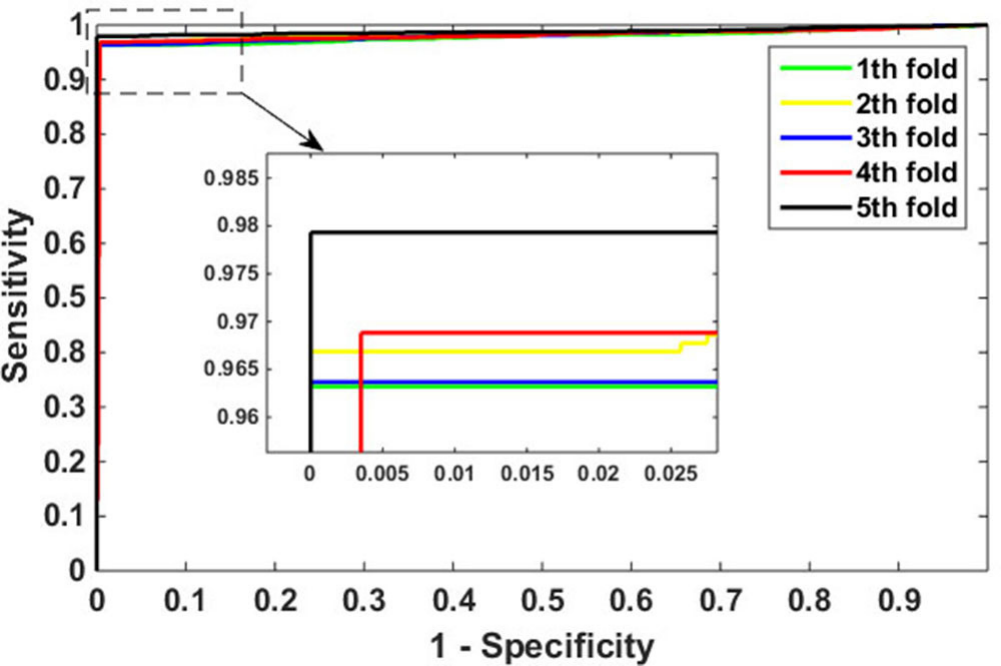
ROC curves performed by proposed method on *Yeast* data set

In this paper, 5-fold cross-validation technique is used as a means to evaluate our model. More specifically, the entire feature data set is randomly divided into five approximately equal subsets. Four of these subsets are used for training and the rest of the subset for testing. The cross-validation process is repeated 5 times so that each data set can be used for testing once. Table 1 lists the results of our predictions on *Yeast* data set, the value of average accuracy, precision, sensitivity, and MCC are *98.38%, 99.92%, 96.84%*, and *96.82%*, respectively. The prediction accuracy of the five models are all greater than *98.17%*, the precisions are greater than *99.62%*, the sensitivities are greater than *96.32%*, and the MCC are greater than *96.40%.* The ROC curves performed on *Yeast* data set is shown in Figure 2. In this figure, X-ray depicts false positive rate (FPR) while y-ray depicts true positive rate (TPR).

**Table 1:** 5-fold cross-validation results obtained by using proposed method on *Yeast* data set

Testing set	Accu.(%)	Prec.(%)	Sen.(%)	MCC(%)
**1**	98.17	100.00	96.32	96.40
**2**	98.30	100.00	96.69	96.66
**3**	98.17	100.00	96.37	96.40
**4**	98.30	99.62	96.88	96.65
**5**	98.97	100.00	97.93	97.97
**Average**	**98.38±0.34**	**99.92±0.17**	**96.84±0.65**	**96.82±0.66**

### The performance of the proposed method on the *H. pylori* data set

**Figure 3:**
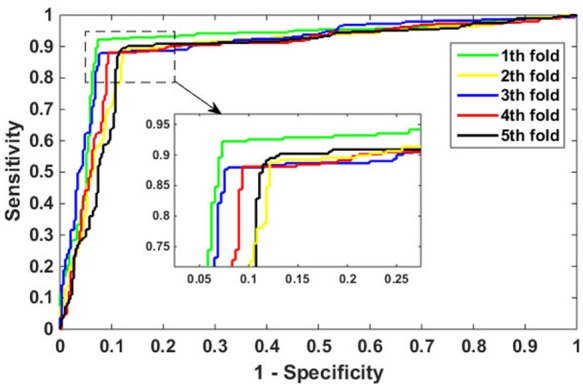
ROC curves performed by the proposed method on *H. pylori* data set

To better evaluate the performance of the proposed model in PPIs prediction, we focused on the testing of *H. pylori* data set. We use the same feature extraction method and the same RF parameters to verify its effect, the results achieved as shown in Table 2. On the *H. pylori* data set we obtain the accuracy of the 5 models are *92.45%, 88.16%, 90.05%, 89.37%*, and *88.70%*, respectively. We can see from Table 2 that the excellent prediction performance of our model with an average precision value of 89.75%, precision value of 90.18%, sensitivity value of 89.12%, and MCC value of 81.62%. Additionally, it can also be seen from Table 2 that the standard deviation of accuracy, precision, sensitivity and MCC is as low as 0.0167, 0.0274, 0.0183 and 0.0269. The ROC curves are shown in Figure 3.

**Table 2:** 5-fold cross-validation results obtained by using proposed method on *H. pylori* data set

Testing set	Accu.(%)	Prec.(%)	Sen.(%)	MCC(%)
**1**	92.45	93.44	92.23	86.00
**2**	88.16	86.93	88.49	79.10
**3**	90.05	92.06	87.63	82.06
**4**	89.37	90.56	88.10	80.99
**5**	88.70	87.93	89.16	79.95
**Average**	**89.75±1.67**	**90.18±2.74**	**89.12±1.83**	**81.62±2.69**

### Comparison with previous method

In recent years, many researchers have proposed various models to predict the PPIs and achieved good results. In order to further evaluate the prediction performance, we compare the proposed method with these excellent methods in the same benchmark data sets. In addition, as the state-of-the-art classification algorithm, SVM has been successfully used to predict PPIs. In this experiment, we also compare the classification performance between Rotation Forest classifier and SVM classifier on the *Yeast* data set. The corresponding parameters of the SVM were selected by the grid search method, and finally we set *c*=0.1 and *g*=0.2, respectively. The LIBSVM tools we adopted are downloaded at www.csie.ntu.edu.tw/~cjlin/libsvm. Table 3 and Table 4 summarize the results of these comparisons.

**Table 3:** Performance comparison of different models on *Yeast* data set

Model	Test set	Accu.(%)	Prec.(%)	Sen.(%)	MCC(%)
Guos’ work [17]	ACC	89.33±2.67	88.87±6.16	89.93±3.68	N/A
	AC	87.36±1.38	87.82±4.33	87.30±4.68	N/A
Zhous’ work [40]	SVM + LD	88.56±0.33	89.50±0.60	87.37±0.22	77.15±0.68
Yangs’ work [41]	Cod1	75.08±1.13	74.75±1.23	75.81±1.20	N/A
	Cod2	80.04±1.06	82.17±1.35	76.77±0.69	N/A
	Cod3	80.41±0.47	81.86±0.99	78.14±0.90	N/A
	Cod4	86.15±1.17	90.24±0.45	81.03±1.74	N/A
Yous’ work [42]	PCA-EELM	87.00±0.29	87.59±0.32	86.15±0.43	77.36±0.44
**Our method**	**SVM+PSSM**	**95.19±0.42**	**94.72±0.68**	**95.72±0.53**	**90.84±0.75**
	**RF + PSSM**	**98.38±0.34**	**99.92±0.17**	**96.84±0.65**	**96.82±0.66**

**Table 4:** Performance comparison of different models on *H. pylori* data set

Model	Accu.(%)	Prec.(%)	Sen.(%)	MCC(%)
Phylogentic bootstrap [43]	75.80	80.20	69.80	N/A
HKNN [44]	84.00	84.00	86.00	N/A
Signature products [30]	83.40	85.70	79.90	N/A
Ensemble of HKNN [45]	86.60	85.00	86.70	N/A
Boosting [46]	79.52	81.69	80.37	70.64
Ensemble ELM [42]	87.50	86.15	88.95	78.13
Our method	**89.75**	**90.18**	**89.12**	**81.62**

Table 3 shows the average prediction results of the different models on the *Yeast* data set, we can see that the accuracy obtained by other methods are between *75.08%* and *89.33%*, the average accuracy obtained by our method is *98.38%*. In the comparison of classifiers, the accuracy obtained on the rotation forest classifier is higher than those obtained on the support vector machine classifier. Table 4 shows the performance of different methods on the *H. pylori* data sets. We can see from the Table 4 that the accuracies of the other six methods are 75.80%, 84.00%, 83.40%, 86.60%, 79.52% and 87.50%, while our method is 89.75%; the precisions of the other six methods are 80.20%, 84.00%, 85.70%, 85.00%, 81.69% and 86.15%, while our method is 90.18%; the sensitivity of the other six methods are 69.80%, 86.00%, 79.90%, 86.70%, 80.37% and 88.95%, while our method is 89.12%. The results obtained by these methods are significantly lower than ours.

### Performance on independent data sets

After completing the experiment on the *Yeast* and *H. pylori* data sets, we continue to test the performance of the proposed method on the *independent* data sets (*C.elegans, E.coli, H. sapiens and M.musculus*). In the experiment, we take all the *Yeast* data set as training set, *independent* data sets as the test set to predict protein-protein interactions. Table 5 lists the accuracy of our method on four data sets. It can be seen from the table that the highest accuracy of the proposed method is 98.50% on the *C.elegans* data set, and even the lowest accuracy achieved on the *E.coli* data set reached 91.00%. It is demonstrates that our method has good accuracy in predicting the interaction of other species.

**Table 5:** Prediction results on four species based on our model

Species	Test pairs	Accu.(%)
***C.elegans***	4013	98.50
***E.coli***	6954	91.00
***H.sapiens***	1412	97.45
***M.musculus***	313	98.08

### Validate potential protein-protein interactions from the PPIs database

After evaluating the effectiveness of the proposed model by using the 5-fold cross validation method, we here calculate the interaction probability for all potential protein-protein pairs in the datasets of *Yeast*. Specifically, the whole negative and positive data explored in 5-fold cross validation experiments are used for training and all the unknown protein-protein pairs are used as testing set. The predicted protein pairs with top-100 ranks in the potential PPI lists are considered as highly potential protein-protein interactions and further verified by three public databases (i.e. DIP [47], MINT [48] and IntAct [49]). These databases have been supplemented by some newly detected protein-protein interactions since the gold standard data explored in this study were collected in 2007. All the predicted possibilities for top 100 potential PPIs in *Yeast* can be obtained in Supplementary Table S1. As shown in Table 6, 15 new protein-protein interactions are finally confirmed. Note that the high-ranked interactions that are not reported yet may also exist in reality. Based on these results, we anticipate that the proposed model is feasible to predict new protein-protein interactions.

**Table 6:** The newly confirmed PPIs with high possibility in the *Yeast* data set

Protein ID	Protein ID	The probability of protein-protein interactions	Evidence
DIP:1113N	DIP:655N	0.9917	DIP
sw:P29295	sw:P20604	0.9912	MINT
sw:P47054	sw:P49687	0.9908	IntAct
DIP:1040N	DIP:2463N	0.9891	DIP
sw:P04050	sw:P16370	0.9869	MINT
DIP:2808N	DIP:6282N	0.9854	DIP
DIP:1408N	DIP:6416N	0.9848	DIP
DIP:1558N	DIP:2370N	0.9846	DIP
DIP:5037N	DIP:799N	0.9840	DIP
sw:Q12176	sw:Q03532	0.9839	MINT, IntAct
DIP:1364N	DIP:2483N	0.9836	DIP
DIP:1726N	DIP:834N	0.9833	DIP
DIP:2417N	DIP:5630N	0.9831	DIP
sw:P18888	sw:P32591	0.9826	MINT, IntAct
sw:Q04067	sw:P40217	0.9812	MINT, IntAct

## MATERIALS AND METHODS

### Data sources

We evaluate our model focus on publicly available *Saccharomyces cerevisiae* data set introduced by Guo *et al.* [17]. The PPIs data were extracted from *Saccharomyces cerevisiae* core subset of database of interacting proteins (DIP) [47], version DIP_20070219. Through the two algorithms, paralogous verification method (PVM) and expression profile reliability (EPR) [50], the core subset of reliability is tested. And less than 50 residues of the protein of protein pairs are removed. In order to reduce pairwise sequence redundancy, multiple sequence alignment tool, CD-Hit [51, 52], was adopted with a threshold of 40% identity. Eventually the 5594 proteins are left to form the positive data set. The negative dataset consists of 5594 additional protein pairs, which are selected at different subcellular localization. Therefore, the positive and negative data set each accounted for half of the 11188 protein pairs constitute the final data set.

As a comparison, we further assess the capabilities of our model in the *H. pylori* data set, which was described by Rain *et al.* [53]. It can be downloaded at http://www.cs.sandia.gov/~smartin/software.html. This data set contains 2916 protein pairs which include half interacting pairs and half non-interacting pairs. It provides a platform for comparing different methods [30, 42, 43, 45, 46].

### Position-specific scoring matrix

Position-Specific Scoring Matrix (PSSM) is used to detect the distantly related proteins, and initially introduced by Gribskov *et al.* [32]. It has made outstanding achievements in these areas: protein secondary structure prediction [54], prediction of disordered regions [55], and protein binding site prediction [56]. A PSSM is an L *×* 20 matrix, which can be denoted as $PSSM=\left\{a_{i,j}:i=1\cdots L\;and\;j=1\cdots 20\right\}$, where L denotes protein sequence length and the number of *20* is due to 20 amino acids. Each element *PSSM (i, j)* of the matrix is defined as follows:

$$PSSM=\begin{array}{c} \begin{array}{cc} a_{1,1} & a_{1,2} \end{array}\;\begin{array}{cc} \cdots & a_{1,20} \end{array}\\ \begin{array}{cc} a_{2,1} & a_{2,2} \end{array}\;\begin{array}{cc} \cdots & a_{2,20} \end{array}\\ \begin{array}{cc} \vdots \; & \vdots \; \end{array}\;\begin{array}{cc} \vdots \; & \vdots \end{array}\\ \begin{array}{cc} a_{L,1} & a_{L,2} \end{array}\;\begin{array}{cc} \cdots & a_{L,20} \end{array} \end{array}$$ (5)

where *α_i,j_* in the *i* row of PSSM means that the probability of the *i*th residue being mutated into type *j* of *20* native amino acids during the procession of evolutionary in the protein from multiple sequence alignments.

In order to extract the evolutionary information, each protein sequence in the data set is used to align and search homogenous sequences from *SwissProt* database by the Position Specific Iterated BLAST (PSI-BLAST) [57] tool. PSI-BLAST will return a 20-dimensional vector which indicates the probabilities of conservation against mutations to 20 different amino acids including its own. To get broad and high homologous sequences, we select in this study the value of e-value is *0.001* and the value of iterations is *3*, respectively. Applications of PSI-BLAST and *SwissProt* database can be downloaded at http://blast.ncbi.nlm.nih.gov/Blast.cgi.

### Pseudo position-specific score matrix

In order to reduce the probability of missing sequence-order information, we introduced the concept of pseudo amino acid composition by Chou *et al.* [58]. In this article the sample of a protein sequence PSSM is represented by Equation 5 and PsePSSM obtained from the following Equation:

$$a_{i,j}=\frac{a_{i,j}^{0}\;-\;\frac{1}{20}\sum_{k=1}^{20}a_{i,k}^{0}}{\sqrt{{\frac{1}{20}\sum_{u=1}^{20}a_{i,u}^{0}-\frac{1}{20}\sum_{k=1}^{20}a_{i,k}^{0}}^{2}}}\;i=1\ldots 20,j=1\ldots 20$$ (6)

where $a_{i,j}^{0}$ represents the original scores directly generated by the PSI-BLAST, and its value is typically positive integers or negative integers. This is not what we want standardized scores, which may have zero means if more than 20 amino acids and may remain unchanged if it continues through the same conversion program. The positive score implies that the corresponding mutation appears more frequently in the alignment than expected by chance, and the negative score, on the contrary, implies that the corresponding mutation appears less frequently in the alignment than expected by chance. However, according to the definition of PSSM, different lengths of proteins will correspond to different rows number in matrices. Equation 7 is employed to express the protein sample PSSM, so that the PSSM descriptor can be represented as a uniform pattern.

$${\bar{P}}_{PSSM}=\;\left[{\bar{a}}_{1}\;{\bar{a}}_{2}\;\cdots \;{\bar{a}}_{20}\right]\;$$ (7)

and

$${\bar{a}}_{j}=\;\frac{1}{L}\overset{L}{\underset{i=1}{\sum }}a_{i,j}\;j=1..20$$ (8)

where ${\bar{a}}_{j}$ denotes the average score when the amino acid residues in protein *P* in the process of running the algorithm was evolved into amino acid type *j*. However, if only ${\bar{P}}_{PSSM}$ is used to represent the protein *P*, all the sequence information will be lost during evolution. In order to prevent the occurrence of missing all information of sequence-order, the thought of pseudo amino acid was introduced to improve the Equation 7. Hence, based on the Equation 9 segmented PsePSSM features can be obtained:

$${\bar{a}}_{j}=\;\{\begin{array}{c} \frac{1}{L}\overset{L}{\underset{i=1}{\sum }}a_{i,j}\;j=1..20,\;\varepsilon =0\;\\ \frac{1}{L-\varepsilon }\overset{L-\varepsilon }{\underset{i=1}{\sum }}{\left(a_{i,j}-\;a_{i+\varepsilon ,j}\right)}^{2}\;j=1..20,\;\varepsilon <L\; \end{array}$$ (9)

where ${\bar{a}}_{j}$ is the correlation factors of amino acid type *j*. Although the value allowed for *ε* can be 0, 1, 2, …, or 49, considering the time costs and efficiency factors, we took *ε* to 0,1,2,3,4, so a total of 200-dimensional vectors are eventually used in this study.

### Rotation forest

Rotation Forest (RF) is a novel proposed ensemble classifier that uses independently trained decision trees. The main idea of the Rotation Forest simultaneously encourages individual accuracy and diversity within the ensemble. In order to generate the training samples of the base classifier, the feature set is randomly divided into *K* subsets. The linear transformation method is applied to each subset, and retains all the principal components to maintain the precision of data. The rotation formed the training sample of new features to ensure the diversity of data. Hence the rotation forest can enhance the accuracy of individual classifier and the diversity in the ensemble at the same time.

Suppose that $\left\{x_{i},y_{i}\right\}$ contains *N* training samples, where $x_{i}=(x_{i1},x_{i2},\ldots ,x_{iD})$ be a *D*-dimensional feature vector, $X={\left(x_{1},x_{2},⊃,x_{n}\right)}^{T}$ be the training sample set (*n×D* matrix), which is composed of *n* observation feature vector composition, $Y={\left(y_{1},y_{2},\ldots ,y_{n}\right)}^{T}$ be the corresponding labels, and *S* be the feature set. Assuming that the number of decision trees in the rotation forest is L, expressed as $R_{1},R_{2},\ldots ,R_{L}$, respectively, and the feature set is randomly divided into K subsets of equal size. The preprocessing steps for an individual classifier is: the first select the appropriate parameters *K* which is a factor of *n,* and *S* randomly divided into *K* disjoint subsets, so the number of features contained in each feature subset is C=nk; the second from the training dataset *X* to select the corresponding column of the feature in the subset *R_i,j_*, form a new matrix *X_i,j_*. Then the bootstrap subset of objects extracts three-quarters the size of the data set from *X* to construct a new training subset *X^'^_i,j_*; The third matrix *X^'^_i,j_* is used as the feature transform for producing the coefficients in a matrix *M_i,j_*, which *j*th column coefficient as the characteristic component *j*th; and the final a sparse rotation matrix *Mat_i_* is formed, and its coefficients in matrix *M_i,j_* is expressed as Equation 10:

$$Mat_{i}=\begin{array}{cccc} a_{i,1}^{\left(1\right)},\ldots ,a_{i,1}^{\left(C_{1}\right)} & 0 & \cdots & 0\\ 0 & \;a_{i,2}^{\left(1\right)},\cdots ,a_{i,2}^{\left(C_{2}\right)} & \cdots & 0\\ \vdots & \vdots & \ddots & \vdots \\ 0 & 0 & \cdots & a_{i,k}^{\left(1\right)},\ldots ,a_{i,k}^{\left(C_{k}\right)} \end{array}$$ (10)

In the prediction phase, given a test sample *x*, let $d_{i,j}(XMat_{i}^{a})$ be the probability produced by the classifier *R_i_* to the hypothesis in which *x* belongs to class *Y_i_*. Then the confidence for a class can be computed according to the average combined method shown in Equation 11:

$$\mu_{j}\left(x\right)=\frac{1}{L}\overset{L}{\underset{i=1}{\sum }}d_{i,j}(XMat_{i}^{a})$$ (11)

**Figure 4:**
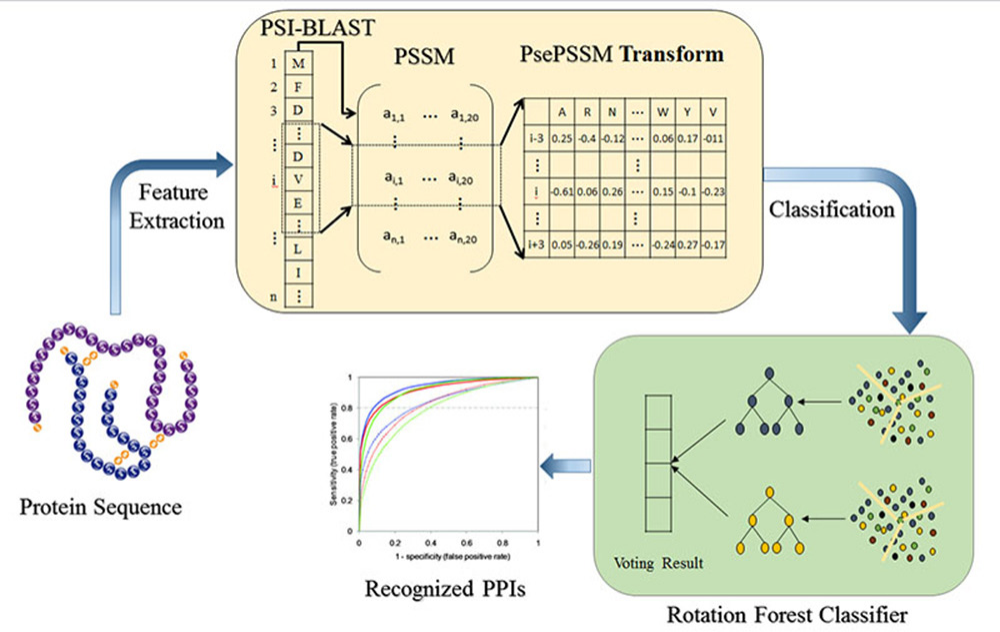
The schematic diagram of the prediction model

Therefore, the test sample *x* easily assigned to the classes with the greatest possible. The schematic diagram of the prediction model is shown in Figure 4.

## CONCLUSIONS

In this paper, we proposed a novel method to predict protein-protein interactions using the Rotation Forest combine with Pseudo Position-Specific Score Matrix. In order to preserve as much information as possible, we first convert the protein amino acids sequences into the PSSM matrix, and then extract the features using the PsePSSM algorithm, finally determine whether there is an interaction between protein pairs through the RF classifier. To evaluate the performance of the proposed method, we implement it on the *Yeast*, *H. pylori* and *independent* data sets. In addition, we also compare the proposed method with other excellent methods. Excellent experimental results demonstrate that the proposed method is feasible and effective in the prediction of protein interactions. The low standard deviation of these criterion values indicates that our method is stable and robust. In future studies, we will focus on improving the classification algorithm to expect higher predictive accuracy and less time consumption in predicting protein-protein interactions.

## WEBSERVER

In order to facilitate the use of researchers, we have built a web server to implement the proposed prediction model. The web server provides the source code and the *Yeast* data sets used in this article for users to download. It can be accessed to at http://202.119.201.126:8888/PsePSSM/. Users can query the predicted results of the *Yeast* data sets through the webpage and receive the predict results by e-mail.

## SUPPLEMENTARY TABLE





## References

[1] Braun P, Gingras A-C (2012). History of protein-protein interactions: From egg-white to complex networks. Proteomics.

[2] Ehrenberger T, Cantley LC, Yaffe MB (2015). Computational prediction of protein-protein interactions. Methods in molecular biology (Clifton, NJ).

[3] Alon U (2003). Biological networks: The tinkerer as an engineer. Science.

[4] Fields S, Song O (1989). A novel genetic system to detect protein-protein interactions. Nature.

[5] Ito T, Chiba T, Ozawa R, Yoshida M, Hattori M, Sakaki Y (2001). A comprehensive two-hybrid analysis to explore the yeast protein interactome. Proceedings of the National Academy of Sciences of the United States of America.

[6] Gavin AC, Bosche M, Krause R, Grandi P, Marzioch M, Bauer A, Schultz J, Rick JM, Michon AM, Cruciat CM, Remor M, Hofert C, Schelder M, Brajenovic M, Ruffner H, Merino A (2002). Functional organization of the yeast proteome by systematic analysis of protein complexes. Nature.

[7] Ho Y, Gruhler A, Heilbut A, Bader GD, Moore L, Adams SL, Millar A, Taylor P, Bennett K, Boutilier K, Yang LY, Wolting C, Donaldson I, Schandorff S, Shewnarane J, Vo M (2002). Systematic identification of protein complexes in Saccharomyces cerevisiae by mass spectrometry. Nature.

[8] Williams NE (2000). Immunoprecipitation procedures. Methods in Cell Biology, Vol 62.

[9] Zhu H, Bilgin M, Bangham R, Hall D, Casamayor A, Bertone P, Lan N, Jansen R, Bidlingmaier S, Houfek T, Mitchell T, Miller P, Dean RA, Gerstein M, Snyder M (2001). Global analysis of protein activities using proteome chips. Science.

[10] Aloy P, Russell RB (2002). Interrogating protein interaction networks through structural biology. Proceedings of the National Academy of Sciences of the United States of America.

[11] Aloy P, Russell RB (2003). InterPreTS: protein Interaction Prediction through Tertiary Structure. Bioinformatics.

[12] Bock JR, Gough DA (2001). Predicting protein-protein interactions from primary structure. Bioinformatics.

[13] Zhang QC, Petrey D, Deng L, Qiang L, Shi Y, Thu CA, Bisikirska B, Lefebvre C, Accili D, Hunter T, Maniatis T, Califano A, Honig B (2012). Structure-based prediction of protein- protein interactions on a genome-wide scale. Nature.

[14] Huang TW, Tien AC, Lee YCG, Huang WS, Peng CL, Tseng HH, Kao CY, Huang CYF (2004). POINT: a database for the prediction of protein-protein interactions based on the orthologous interactome. Bioinformatics.

[15] Espadaler J, Romero-Isart O, Jackson RM, Oliva B (2005). Prediction of protein-protein interactions using distant conservation of sequence patterns and structure relationships. Bioinformatics.

[16] Shen J, Zhang J, Luo X, Zhu W, Yu K, Chen K, Li Y, Jiang H (2007). Predictina protein-protein interactions based only on sequences information. Proceedings of the National Academy of Sciences of the United States of America.

[17] Guo Y, Yu L, Wen Z, Li M (2008). Using support vector machine combined with auto covariance to predict proteinprotein interactions from protein sequences. Nucleic Acids Research.

[18] Zhang Y-N, Pan X-Y, Huang Y, Shen H-B (2011). Adaptive compressive learning for prediction of protein-protein interactions from primary sequence. Journal of Theoretical Biology.

[19] Liu CH, Li K-C, Yuan S (2013). Human protein-protein interaction prediction by a novel sequence-based co-evolution method: co-evolutionary divergence. Bioinformatics.

[20] Gao ZG, Wang L, Xia SX, You ZH, Yan X, Zhou Y (2016). Ens-PPI: A Novel Ensemble Classifier for Predicting the Interactions of Proteins Using Autocovariance Transformation from PSSM. Biomed Research International.

[21] You ZH, Lei YK, Gui J, Huang DS, Zhou XB (2010). Using manifold embedding for assessing and predicting protein interactions from high-throughput experimental data. Bioinformatics.

[22] Lei YK, You ZH, Ji Z, Zhu L, Huang DS (2012). Assessing and predicting protein interactions by combining manifold embedding with multiple information integration. Bmc Bioinformatics.

[23] Luo X, You ZH, Zhou MC, Li S, Leung H, Xia YN, Zhu QS (2015). A Highly Efficient Approach to Protein Interactome Mapping Based on Collaborative Filtering Framework. Scientific Reports.

[24] You Z-H, Zhou M, Luo X, Li S (2016). Highly Efficient Framework for Predicting Interactions Between Proteins.

[25] Huang Y-A, You Z-H, Li X, Chen X, Hu P, Li S, Luo X (2016). Construction of reliable protein–protein interaction networks using weighted sparse representation based classifier with pseudo substitution matrix representation features. Neurocomputing.

[26] Ben-Hur A, Noble WS (2005). Kernel methods for predicting protein-protein interactions. Bioinformatics.

[27] Xu Y, Hu W, Chang Z, DuanMu H, Zhang S, Li Z, Li Z, Yu L, Li X (2011). Prediction of human protein-protein interaction by a mixed Bayesian model and its application to exploring underlying cancer-related pathway crosstalk. Journal of the Royal Society Interface.

[28] Saha I, Zubek J, Klingstrom T, Forsberg S, Wikander J, Kierczak M, Maulik U, Plewczynski D (2014). Ensemble learning prediction of protein-protein interactions using proteins functional annotations. Molecular Biosystems.

[29] Yang L, Tang X (2014). Protein-Protein Interactions Prediction Based on Iterative Clique Extension with Gene Ontology Filtering. Scientific World Journal.

[30] Martin S, Roe D, Faulon JL (2005). Predicting protein-protein interactions using signature products. Bioinformatics.

[31] Ahmad S, Sarai A (2005). PSSM-based prediction of DNA binding sites in proteins. Bmc Bioinformatics.

[32] Gribskov M, McLachlan AD, Eisenberg D (1987). Profile analysis: detection of distantly related proteins. Proceedings of the National Academy of Sciences of the United States of America.

[33] Chou K-C, Shen H-B (2007). MemType-2L: A Web server for predicting membrane proteins and their types by incorporating evolution information through Pse-PSSM. Biochemical and Biophysical Research Communications.

[34] Nanni L, Lumini A, Brahnam S (2013). An empirical study on the matrix-based protein representations and their combination with sequence-based approaches. Amino Acids.

[35] Shen H-B, Chou K-C (2007). Nuc-PLoc: a new web-server for predicting protein subnuclear localization by fusing PseAA composition and PsePSSM. Protein Engineering Design & Selection.

[36] Rodriguez JJ, Kuncheva LI (2006). Rotation forest: A new classifier ensemble method. Ieee Transactions on Pattern Analysis and Machine Intelligence.

[37] Nanni L, Lumini A (2009). Ensemble generation and feature selection for the identification of students with learning disabilities. Expert Systems with Applications.

[38] Zweig MH, Campbell G (1993). Receiver-operating characteristic (ROC) plots: a fundamental evaluation tool in clinical medicine. Clinical chemistry.

[39] Chatfield C (2004). Statistical data mining and knowledge discovery. Journal of the Royal Statistical Society Series a-Statistics in Society.

[40] Zhou YZ, Gao Y, Zheng YY (2011). Prediction of Protein-Protein Interactions Using Local Description of Amino Acid Sequence. Advances in Computer Science and Education Applications, Pt Ii.

[41] Yang L, Xia J-F, Gui J (2010). Prediction of Protein-Protein Interactions from Protein Sequence Using Local Descriptors. Protein and Peptide Letters.

[42] You Z-H, Lei Y-K, Zhu L, Xia J, Wang B (2013). Prediction of protein-protein interactions from amino acid sequences with ensemble extreme learning machines and principal component analysis. Bmc Bioinformatics.

[43] Bock JR, Gough DA (2003). Whole-proteome interaction mining. Bioinformatics.

[44] Nanni L (2005). Hyperplanes for predicting protein-protein interactions. Neurocomputing.

[45] Nanni L, Lumini A (2006). An ensemble of K-local hyperplanes for predicting protein-protein interactions. Bioinformatics.

[46] Liu B, Yi J, Aishwarya SV, Lan X, Ma Y, Huang THM, Leone G, Jin VX (2013). QChIPat: a quantitative method to identify distinct binding patterns for two biological ChIP-seq samples in different experimental conditions. Bmc Genomics.

[47] Xenarios I, Salwinski L, Duan XQJ, Higney P, Kim SM, Eisenberg D (2002). DIP, the Database of Interacting Proteins: a research tool for studying cellular networks of protein interactions. Nucleic Acids Research.

[48] Chatr-aryamontri A, Ceol A, Palazzi LM, Nardelli G, Schneider MV, Castagnoli L, Cesareni G (2007). MINT: the molecular INTeraction database. Nucleic Acids Research.

[49] Kerrien S, Alam-Faruque Y, Aranda B, Bancarz I, Bridge A, Derow C, Dimmer E, Feuermann M, Friedrichsen A, Huntley R, Kohler C, Khadake J, Leroy C, Liban A, Lieftink C, Montecchi-Palazzi L (2007). IntAct - open source resource for molecular interaction data. Nucleic Acids Research.

[50] Deane CM, Salwinski L, Xenarios I, Eisenberg D (2002). Protein interactions - Two methods for assessment of the reliability of high throughput observations. Molecular & Cellular Proteomics.

[51] Li WZ, Jaroszewski L, Godzik A (2001). Clustering of highly homologous sequences to reduce the size of large protein databases. Bioinformatics.

[52] Li W, Godzik A (2006). Cd-hit: a fast program for clustering and comparing large sets of protein or nucleotide sequences. Bioinformatics.

[53] Rain JC, Selig L, De Reuse H, Battaglia V, Reverdy C, Simon S, Lenzen G, Petel F, Wojcik J, Schachter V, Chemama Y, Labigne AS, Legrain P (2001). The protein-protein interaction map of Helicobacter pylori. Nature.

[54] Jones DT (1999). Protein secondary structure prediction based on position-specific scoring matrices. Journal of molecular biology.

[55] Jones DT, Ward JJ (2003). Prediction of disordered regions in proteins from position specific score matrices. Proteins-Structure Function and Bioinformatics.

[56] Chen X-W, Jeong JC (2009). Sequence-based prediction of protein interaction sites with an integrative method. Bioinformatics.

[57] Altschul SF, Madden TL, Schaffer AA, Zhang J, Zhang Z, Miller W, Lipman DJ (1997). Gapped BLAST and PSI-BLAST: a new generation of protein database search programs. Nucleic acids research.

[58] Chou KC (2001). Prediction of protein cellular attributes using pseudo-amino acid composition. Proteins-Structure Function and Genetics.

