# Supplementary material for: An ensemble approach for large-scale identification of protein-protein interactions using the alignments of multiple sequences
Source: Oncotarget. 2016 Dec 22;8(3):5149–59. doi: 10.18632/oncotarget.14103 (PMC5354898; doi:10.18632/oncotarget.14103)
Supplement: Supplementary file 1 [file oncotarget-08-5149-s001.pdf]

An ensemble approach for large-scale identification of protein-protein interactions using the alignments of multiple sequences

SUPPLEMENTARY TABLE

Supplementary Table S1: We implemented the proposed method on Yeast data set for potential protein-protein interactions. As a result, 15 out of the top 100 predicted PPIs were confirmed by the databases of DIP, MINT and IntAct

See Supplementary File 1
